# Evapotranspiration Cycles in a High Latitude Agroecosystem: Potential Warming Role

**DOI:** 10.1371/journal.pone.0137209

**Published:** 2015-09-14

**Authors:** Watcharee Ruairuen, Gilberto J. Fochesatto, Elena B. Sparrow, William Schnabel, Mingchu Zhang, Yongwon Kim

**Affiliations:** 1 School of Natural Resources and Extension, University of Alaska Fairbanks, Fairbanks, Alaska, United States of America, 99775; 2 Department of Atmospheric Sciences, Geophysical Institute and College of Natural Science and Mathematics, University of Alaska Fairbanks, Fairbanks, Alaska, United States of America, 99775; 3 Water and Environmental Research Center, Institute of Northern Engineering, University of Alaska Fairbanks, Fairbanks, Alaska, United States of America, 99775; 4 International Arctic Research Center, University of Alaska Fairbanks, Fairbanks, Alaska, United States of America, 99775; The Ohio State University, UNITED STATES

## Abstract

As the acreages of agricultural lands increase, changes in surface energetics and evapotranspiration (ET) rates may arise consequently affecting regional climate regimes. The objective of this study was to evaluate summertime ET dynamics and surface energy processes in a subarctic agricultural farm in Interior Alaska. The study includes micrometeorological and hydrological data. Results covering the period from June to September 2012 and 2013 indicated consistent energy fractions: *LE*/*R*_*net*_ (67%), *G*/*R*_*net*_ (6%), *H*/*R*_*net*_ (27%) where *LE* is latent heat flux, *R*_*net*_ is the surface net radiation, *G* is ground heat flux and *H* is the sensible heat flux. Additionally actual surface evapotranspiration from potential evaporation was found to be in the range of 59 to 66%. After comparing these rates with those of most prominent high latitude ecosystems it is argued here that if agroecosystem in high latitudes become an emerging feature in the land-use, the regional surface energy balance will significantly shift in comparison to existing Arctic natural ecosystems.

## Introduction

Recent warming in high-latitudes has significantly impacted Alaska’s ecosystems [1–2]. These impacts have affected a broad spectrum of ecological, physical and societal systems of the Arctic [2–9]. In this context, agroecosystems and food related economic activities may be highly impacted by climate change over the next decades [10–11]. Therefore in order to meet future demands and conduct sustainable agriculture, considerable increase in food production must reduce the environmental impact [12].

Agroecosystems in Alaska currently represent only a small fraction of the entire landscape consisting mainly of boreal forests and tundra. Two current trends are supporting re-invigoration of Alaskan agriculture. First, desire for local food production and concerns about food security. Second, the combination of lengthening of the growing season, higher surface temperatures and greater precipitation rates [2, 13–17] would enhance the regional agricultural capacity [18, 19]. To substantiate these trends, the expected changes at the end of the current century in mean surface temperature will range from 1.5 to 4.5°C [20]. Summer warming in the Alaskan Arctic has been observed to accelerate at rates from 0.3°C to 0.4°C per decade [2] peaking in the snow-free season (0.4°C to 0.6°C per decade) [3, 21–23]. As a consequence of this warming trend, Arctic and subarctic areas are experiencing longer growing seasons which in turn favor the implementation of large scale agriculture, as indicated by Juday et al. [18] and Hatch [19], albeit not in all areas [24].

According to future scenarios of growing degree days [18–19], favorable conditions for developing agricultural lands may also expand crop variety (e.g., cash crops such as corn or canola). However, there are major environmental challenges in high latitude settings that may have a counteracting influence on sustainable agriculture and expansion. Some of such elements are strong seasonal variation, cold soils, unpredictable frosts and precipitation events [25–27] and low amounts of accumulated heat energy throughout the growing season [28]. Nevertheless, Interior of Alaska provides a unique growing region that combines atmospheric radiation, warm air temperatures, agricultural and natural resources and water availability.

Soil surveys in Alaska indicate that more than 16 million hectares are suitable for agriculture, where the largest and more productive areas are localized in the Interior along the Tanana River Valley [18, 29]. Hence, Alaska may become more attractive as agriculture in the contiguous US becomes threatened (e.g., increasing droughts). However, if agricultural demands in Alaska were to increase, then part of the boreal forest would be at risk of being cleared for agricultural production purposes. Such land-use change would likely have an important effect on surface-energy balance as well as in water cycling which would potentially lead to local and regional climate changes and feedbacks [30–34]. Specifically this conversion would alter the seasonal albedo, surface roughness, moisture fluxes, and leaf area index [34]. Associated with an increase in air temperature and the lengthening of growing season, agricultural production could be limited by water availability and requirements for irrigation. This in turn would drive land surface-climate interactions by artificially modifying surface water and energy budgets [35–38]. Several observational and modeling studies have shown these effects on both ET and other atmospheric variables. The effects of irrigation are not only restricted to increase of ET over irrigated land [39–40], but also increase of cloud formation, surface cooling and precipitation in nearby non-irrigated areas, and potentially induce changes in mesoscale circulation [41–43]. In Alaska, however, the presence of agricultural land and its potential influence or feedback to regional climate is still unknown.

Evidence of changes in surface energy fluxes [44] and water balance [45] in Arctic ecosystems has been already documented. However, to the authors’ knowledge, the case of agroecosystem in high latitude has not yet been systematically assessed. Here we provide measurements of surface energy balance over two growing seasons in summer of 2012 and 2013 from an agricultural land in Interior Alaska. Previous estimates of potential ET and actual ET have been carried out by other researchers in the same site using various methods [46–48]. They found annual values of potential ET to be in the range 360 to 467 mm for Fairbanks [46–47]. As for the growing season (14 June to 31 August), the total ET of 223 mm for irrigated barley field, 113 mm for non-irrigated barley field, and 110 mm for fallow field were reported from experiments in the same site of the present study [48].

The objectives of this study are to determine the seasonal cycle of ET and to examine the energy fractioning in high latitude agroecosystem. A comparative assessment is then provided against representative natural ecosystems to highlight the importance and potential influence on climate projections. This information may be important to understand future possibilities for sustainable agricultural, local and regional climate change and feedbacks in the regional climate.

## Materials and Methods

### Experimental Site

**Fig 1:**
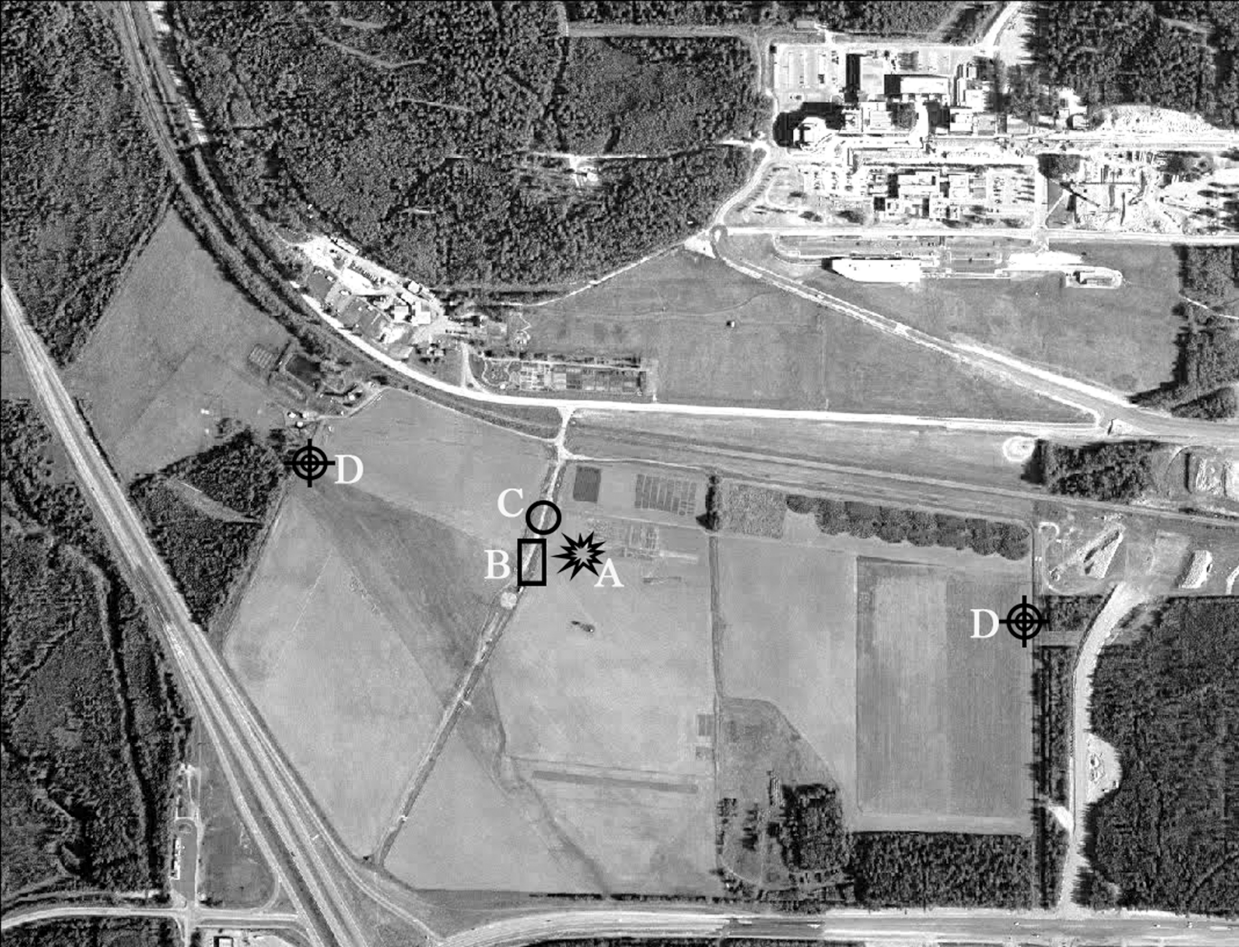
Fairbanks Experiment Farm (FEF) site at the UAF AFES. The location of the instrumentation is illustrated with a different pattern. The farm dimensions are: more than 1 kilometer on East to West direction and about 600 m North to South. EC tower (A), lysimeter plot (B), Meteorological station (C), LAS system (D). Airborne survey photo was provided by the UAF Department of Design and Construction obtained by AeroMap Inc summer of 2003.

The field experiment was conducted at the Fairbanks Experiment Farm (FEF) on West Tanana Drive of the University of Alaska Fairbanks (UAF) Agricultural and Forestry Experiment Station (AFES), in Fairbanks, Alaska, USA (64° 51′ 16.6″ N, 147° 51′ 36.4 ″ W, 150 m above sea level) (Fig 1). Experimental data were collected during the summer season from June to September of 2012 and 2013. The length of the growing season in the subarctic can be defined as the number of days between the last frost of spring and the first frost of fall. In this period of time the air temperature never drops below the freezing point [49]. Based on meteorological data covering the period 1906–2006, the length of the growing season in Interior of Alaska has increased over the last century by about 45% from 85 to 123 days [50].

The experimental site is characterized by an almost flat topography of the valley floor of the Chena and Tanana River basin. The area is sheltered on three sides from the northwest to the northeast by nearby hills rising to an elevation of 300–500 m, with another barrier about 250 km south the Alaska Range [51–52]. The site has three main vegetation types: woodland, grassland and crops, combined with bare land. The research area has a continental subarctic climate with long, cold winters and short, warm summers. Summer comprises the months of June, July, and August where air temperature average 15°C. On the other hand, winter months of November through March have average air temperatures of -18°C. Considering the central months of the two major seasons, the thirty-year (1981–2010) average air temperature in Fairbanks for July was 22°C with extreme temperatures rising above 32°C and decreasing down to -40°C in the month of December with continuous snow cover ground [53–54]. Precipitation is relatively low with the average annual accumulation for the period 1981–2010 about 263 mm, which mostly occurs in summer months of July and August [54]. In general, day length during the summer month rises up to 22 hours, which leads to a swing in temperature above 27°C for around 13 days. The range of frost free days, (i.e. air temperatures above 0°C) is approximately from 86 to 144 days with a median value of 115 days [54].

Approximately one hundred years ago the research site was part of the Alaskan boreal forest comprised mainly of *Picea mariana* specie and was cleared to be cultivated after 1906. In the past, large quantities of manure have been used as a supplement nutrient [27]. The land had long term tillage and crop residue management practices [55]. The site contains alluvial soil in the flood plains of the Tanana River. It is classified as a Tanana silt loam [53] with an approximate composition of 70% of silt, 8% of clay, and 22% of sand that has been conventionally farmed for about 80 years. It also contains a relatively high concentration of calcium carbonate and calcium sulfate at the soil surface [27]. A perched water table above the permafrost is about 8 m deep while the main water table is located about 20 m deep [27]. Crops were planted into the soil that had been summer fallowed the previous season. Some vegetable crops are usually grown with irrigation to improve and control crop growth allowing better use of the available plant nutrients [53]. As mentioned earlier this site was utilized by Braley [48] to estimate rates of ET from barley *(Hordeum vulgare L)* and rapeseed (*Brassica rapa*) fields during 1978 and 1979.

The research site for this study is considered a baseline for Interior Alaska agricultural research under the UAF AFES FEF and considered to be representative of floodplain Interior Alaska growing condition.

### Instrumentation

#### Lysimeter setup

The experiment deployed several drainage lysimeters (S1 Fig) during the two growing seasons in 2012 and 2013 (June to September). Each lysimeter was built 62 cm long, 62 cm wide and 62 cm high. A lawn mix soil consisting mostly of sandy loam (66% sand), 29% silt, and 5% clay was added to each lysimeter up to 10 cm from the top of each lysimeter prior to the summer of 2012. The lysimeters were installed on a flat land area over a leveled horizontal plane. The bottom of each lysimeter had a 15 cm filter layer that consisted of stones, gravel and sand with a layer of geofabric above and beneath. The geofabric separates the soil from the filter layer. The filter layer kept the soil from spilling into the drainage and helped to drain water during heavy rains or irrigation events. A pipe collected the drainage from the lysimeter bottom.

In 2012, the experiment in lysimeter plots was conducted from 6 June to 16 September. A set of six drainage lysimeters were used to measure ET for lettuce (*Lactuca sativa*) after direct seeding on 8 June 2012. Sensors for soil volumetric moisture content (θ_ly_) in the root zone were deployed at 15 and 30 cm depth in the lysimeters with a sample rate of 1 minute and record interval of 1 hour (S1 Table). Soil temperature at 15 and 30 cm depths was measured with sensors in the lysimeter (S1 Fig). The lysimeters were irrigated throughout the 2012 growing season. The observations were complemented by a multilevel 1, 2, 3 and 5 m meteorological observations, along with measurements of net radiation (R_net_), turbulent velocities (u, v, w), and sonic temperature (T_sonic_) operating from 16 July to 9 September 2012. Additionally, soil volumetric moisture content under barley, brome grass *(Bromus inermis* Leyss.*)*, bare field (θ_FEF_) and soil temperature (T_soil_) at 15 cm depth were measured from 1 June to 30 September at the experiment site.

The intensive period of measurements during summer 2013 was from 14 June to 16 September, with two treatments in which ET was measured. The plot treatments (three replicates) were: (i) vegetated lysimeter and (ii) unvegetated lysimeter. A total of three soil moisture sensors were installed at 5, 10 and 20 cm depths in the vegetated lysimeters (θ_ly_) and unvegetated lysimeters (θ_unly_) treatments (S1 Fig). The soil temperature was measured at 5 and 10 cm depth in each lysimeter. Oak leaf lettuce (*Lactuca sativa*) at the five to six-leaf stage was transplanted into vegetated lysimeters on 1 June 2013. Irrigation was done throughout the 2013 growing season in both treatments by adding the same amount of water to each lysimeter. Irrigation amount ranged from 5.5 mm to 20 mm. Data from measurements in both lysimeter types were used three weeks after set up to allow the soil to settle. Observations during summer 2013 incorporated turbulent flux measurements derived from 3 m high sonic anemometer tower (operating from 7 July to 11 September 2013) and a large aperture scintillometer (LAS), which was operated from 7 July to 30 August. These observations were complemented by soil measurements (T_soil_) in barley, brome grass and bare plots at 15 cm depth (operating from 1 June to 17 September) (S1 Table). All soil moisture and temperature profiles were recorded on dedicated data loggers (S2 Table).

#### Micrometeorological instrumentation

An eddy covariance (EC) instrument and meteorological station (Met station) were installed at the research site (Fig 1 and S1 Table). The EC instrument was placed on a tripod in the center of the farmland. The instrumentation consisted of a three-dimensional (3D) sonic anemometer (RMYoung 81000) mounted at a height of 3 m to measure the three turbulent components of the wind flow vector (u, w, v) with two temperature probes (T_air_) mounted at 1 m and 3 m above the ground to determine air temperature (S2 Table). Data were collected at 20 Hz frequency and fluxes were calculated for a 30-min eddy-covariance average period. With the aim to foster further studies a LAS was also installed on site (Fig 1). ***R***_***net***_ sensor was mounted at 3 m oriented to the south to avoid shade at all times. A barometer (P) was placed at the surface to determine the ambient air pressure. All data sensors were centralized in a single data logger (S2 Table). Additionally, two meteorological stations were mounted at 2 and 5 m above the ground surface to measure air temperature, relative humidity (RH), air pressure, wind speed (U), wind direction, and precipitation at 1-minute sampling rate. Data redundancy ensured a fairly continuous rate of data collection.

#### Pan Evaporation

A standard weather bureau Class A evaporation pan (PE) (122 cm diameter by 25 cm height), located 5 m away from the lysimeter plots, was used to measure manually (hook gage) and determine daily time series of potential evaporation (E_P_). The water level in the pan was maintained within 7.5–12.5 cm of the lip. The evaporation pan is made of aluminum and rests on a wooden platform 12 cm above the ground over non-irrigated grass around the area. Daily Ep measurements were collected at 0800 AM AKST systematically every-day from 20 June to 5 September 2013 and corrected by wind observations atop the pan evaporation (S1 Table).

### Surface Energy Balance

The surface energy balance is established based on Eq 1. As described previously the FEF sits on an almost flat surface terrain with no aerodynamic obstacles on the central section of the farm covering 1 km east-west and approximately 700 m north-south direction.

$$R_{net}-G=H+LE+Res$$ Eq 1

where *R*_*net*_ is the surface net radiation flux (W m^-2^), *G* is conductive ground heat flux (W m^-2^), *H* is the sensible heat flux (W m^-2^), *LE* is the latent heat flux (W m^-2^) and *Res* is the residual closure component. In this case no storage term is considered since the vegetative canopy is very simple.

In Eq 1 the net radiation term *R*_*net*_, was measured directly. Ground heat flux (*G*) was calculated based on soil thermistors over different vegetation covers such as bromegrass field, barley field and fallow field. Soil temperature depths were 5 and 15 cm in summer 2012 experiment and 5, 15, 20 and 30 cm for the summer 2013. The conductive heat flux on the ground was calculated as follows:

$$G=-k.\frac{\partial T}{\partial z}$$ Eq 2

where *T*(*z*) is the soil temperature profile (°C) at specified depths z (cm) and *k* is the soil thermal conductivity. The *k* value in this study is treated as constant at 0.9 W (m°C)^-1^ [54]. The *H* component was measured based on meteorological data and compared to eddy covariance procedure. The latent heat term (*LE*) was estimated using ET method described in following subsections.

Energy balance partitioning is used to determine the total available energy at the surface among the energy balance components by calculating the ratios *LE*/*R*_*net*_, *H*/*R*_*net*_ and *G*/*R*_*net*_. These ratios indicated the relative magnitudes of *LE*, *H* and *G* in the surface energy balance. The ratio of *H*/*LE* flux is the Bowen ratio (β).

### Energy balance closure

Based on independent measurements and determinations of *R*_*net*_, *LE*, *H* and *G*, the surface energy balance was established. Since Eq 1 combines radiative fluxes with turbulent fluxes averaged in space and time; still an energy closure was estimated characterizing the site in terms of the surface-atmosphere interactions. The closure fraction *C*_*F*_ was therefore deduced based on Eq 3.

$$C_{F}=\frac{LE+H}{R_{net}-G}$$ Eq 3

### Estimation of evapotranspiration

#### Penman-Monteith

A number of approaches can be used to estimate ET based on energy balance measurements Penman-Monteith [56, PM hereafter], Priestley-Taylor [57] and Bowen ratio energy balance method [58–59]. Of these, the PM method is the more widely used in advanced ET models [60]. This method estimates ET on the basis of surface aerodynamic properties and physiological characteristics of vegetation. Variables used in the PM method are net radiation, soil heat flux, air temperature, relative humidity, wind speed, and environment-specific variables related to vegetation cover. The aerodynamic and physiological properties of the vegetation known as canopy resistance are the two important factors in the PM model. An approximation to actual evapotranspiration is the modified PM equation with the addition of the surface canopy resistance [61–62]. Several authors have shown the application of PM equation, including the canopy resistance, in a variety of environments. They have also tested this formulation including water stress, over several crops such as grass, lettuce, soybean, cattails, maize, tomato, wheat, and cotton [61–65]. Sensitivity of PM equation to different input data and parameters shows an effective dependence on the aerodynamic and canopy resistance introduced by considering the influence of vegetation type into the equation (see Eq 4)[66].

$$\lambda ET=\frac{\Delta \left(R_{net}-G\right)+\rho_{a}C_{p}\left(e_{s}-e_{a}\right)/r_{a}}{\Delta +\gamma \left(1+\frac{r_{c}}{r_{a}}\right)}$$ Eq 4

where *ET* is the latent heat flux of evapotranspiration (mm h^-1^ or mm day^-1^), *λ* is latent heat of vaporization (kJ kg^-1^), Δ is the slope of saturation vapor pressure versus temperature curve (kPa °C^-1^), *R*_*net*_ is net radiation flux (W m^-2^), *G* is ground heat flux (W m^-2^), *ρ*_*a*_ is the air density (kg m^-3^), *C*_*p*_ is the air mass specific heat (kJ kg^-1^°C^-1^) at constant pressure, *e*_*s*_ is the saturation vapor pressure at ambient air temperature (k Pa), *e*_*a*_ is the actual vapor pressure of the air mass (k Pa), *e*_*s*_ – *e*_*a*_ is the vapor pressure deficit (VPD) (kPa), *γ* is the psychometric constant (kPa °C^-1^), *r*_*a*_ is the aerodynamic resistance (s m^-1^), and *r*_*c*_ is the bulk canopy resistance (s m^-1^). As indicated in Eq 4 the PM method requires information on net radiation, air temperature, air humidity, wind speed, and ground heat flux that can be obtained and deduced from meteorological and radiation observations. In this case, the vapor pressure deficit (VPD; *e*_*s*_ – *e*_*a*_) can be calculated as a function of measured air temperature and relative humidity using Eq 5 and Eq 6.

$$e_{s}\left(T\right)=0.6108exp\left(\frac{17.27\;T}{T+237.3}\right)$$ Eq 5

$$e_{a}=e_{s}\left(T\right)\frac{RH}{100}$$ Eq 6

where *T* is air temperature (°C) and *RH* is relative humidity in (%)

The slope of the saturation vapor pressure (Δ) curve is also a function of temperature and can be calculated based on Eq 7.

$$\Delta =\frac{4098\left[0.6108exp\left(\frac{17.27\;T}{T+237.3}\right)\right]}{{\left(T+237.3\right)}^{2}}$$ Eq 7

The psychometric constant (*γ*) in Eq (4) is a function of atmospheric pressure (which varies slightly over time and altitude) and is given by Eq 8:

$$\gamma =\frac{C_{p}P}{\varepsilon \lambda }$$ Eq 8

where *C*_*p*_ is the specific heat at constant pressure, equal to 1.013 (MJ kg^-1^°C^-1^), *λ* is the latent heat of vaporization 2.45 (MJ kg^-1^), *ε* is the ratio of molecular weight of water vapor/dry air = 0.622, and *P* is the ambient air pressure (kPa).

The aerodynamic resistance (*r*_*a*_) under neutral conditions is calculated from Eq 9 following Allen et al. [67]:

$$r_{a}=\frac{ln\frac{\left(z_{m}-d\right)}{z_{om}}ln\frac{\left(z_{h}-d\right)}{z_{oh}}}{k^{2}u_{z}}$$ Eq 9

where *r*_*a*_ is aerodynamic resistance (s m^-1^), *z*_*m*_ (m) is height of the wind speed measurements, *z*_*h*_ (m) is the height of temperature and humidity measurement, *k* is von Kármán constant (0.41), *u*_*z*_ (m s^-1^) is wind speed measurement at *z*_*m*_, *d* (m) is zero plane displacement height of wind profile, *z*_*om*_ (m) is roughness parameter for momentum, *z*_*oh*_ (m) is roughness parameter for heat and water vapor. Reference values recommended in the literature are *d* = 2/3*h*_*c*_, where *h*_*c*_ is crop height in meters; *z*_*om*_ is 0.123*h*_*c*_, and *z*_*oh*_ is 0.1 [68].

The stomatal resistance was obtained by measurements with a leaf porometer (S2 Table) in the field-scale to calculate canopy resistance. Following the procedure developed by Irmak et al. [65], we have randomly selected crops to determine the stomatal resistance in the field taking readings of leaves from the most representative vegetation patterns present in the farm (e.g., lettuce, barley, smooth bromegrass). Samples were taken during the midday interval from 1100 to 1400 AKST to determine a stable quantity representative of the central part of the day. Then, combining these data with the determination of the Leaf Area Index (LAI) measured by AccuPAR LP-80 ceptometer (S2 Table) the canopy resistance was determined. The variability range of canopy resistance was from 23 to 150 s m^-1^ with a median of 100 and standard deviation of 55 s m^-1^. Thus, the median of 100 s m^-1^ was then applied into the PM equation to estimate the actual evapotranspiration.

#### Priestley-Taylor coefficient

Priestley and Taylor model (PT) [57] calculates potential ET based on the measurements of equilibrium evapotranspiration via an empirical coefficient α. This coefficient varies according to the surface and vegetation type. A constant value of 1.26 is generally used in landscapes where vegetation cover is almost complete and for saturated surface conditions [57, 69]. The PT equation can be applied for unsaturated water surfaces provided α is adjusted to each condition [70]. The PT model has been shown to provide acceptable accuracy for predicting daily evaporation in Arctic ecosystems if the value of α is known [71]. The equation Eq 10 describes the PT approach.

$$\alpha =\frac{\lambda ET}{\frac{s}{s+\gamma }\left(R_{net}-G\right)}$$ Eq 10

where α is an empirical coefficient relating actual evaporation to equilibrium evaporation; *s* is the slope of the saturation vapor pressure and air temperature curve (kPa °C^-1^); *γ* is the psychrometric constant (kPa °C^-1^); *R*_*net*_ is net radiation (W m^-2^); and *G* is ground heat flux (W m^-2^).

#### Mass balance approach

The ET estimated from lysimeters usually derives from applying the mass balance equation as a closed system as well as measurement of the soil water budget and some meteorological variables. The mass balance method is largely used in agriculture especially in crop productions that use irrigation input [67, 72–74]. The mass balance equation is indicated in Eq 11 [75–76].

$$P+I+C_{r}=ET+D+R\pm \Delta S$$ Eq 11

where *P* is precipitation, *I* is irrigation, *C*_*r*_ is capillary rise, *ET* is evapotranspiration that includes canopy interception or wet canopy evaporation and plant transpiration (i.e. dry canopy transpiration), *D* is drainage, *R* is runoff and, Δ*S* is the change in water storage (all terms expressed in mm) in both the unsaturated and saturated soil zones.

In lysimeter systems, the runoff component *R* is not considered and the capillary rise *C*_*r*_ is assumed to be negligible. The mass balance for the study can thus be expressed according to Eq 12:

P+I−D±ΔS=ET Eq 12

To calculate the lysimeter water storage the devices were divided into different layers for which measurements are available by depth, assuming that each soil moisture sensor was installed in a sampling depth layer within the lysimeter. According to Lewan and Jansson [77] on a similar setup, measurements at 5 cm depth were considered to represent 0–7.5 cm layer; 10 cm depth representing 7.5–15 cm layer, and 20 cm corresponding to 15 cm down to the bottom of the soil profile. Then, the value of the soil water storage was obtained per layer after integrating the volumetric soil water content in the specific depth. The total soil water storage was determined by the sum of the storage in each layer Eq 13

$$S=\overset{D}{\underset{0}{\int }}\theta dz≅\sum \theta \Delta z=\theta D$$ Eq 13

where *S* represents the storage (mm), *θ* the volumetric soil water content (m^3^ m^-3^), and *D* the considered soil depth (mm). The change in soil moisture can be obtained by the change in soil moisture content over depth and time as indicated in Eq 14:

$$\Delta S=\int_{0}^{z}\int_{t_{1}}^{t_{2}}\theta dtdz$$ Eq 14

where Δ*S* is the soil water storage, *θ* is the volumetric soil moisture content (m^3^ m^-3^), *t* is the time, and *z* is depth (cm). Hence, the soil water storage variation profile was determined by the difference between the values of the soil moisture content obtained in the final and initial time of each considered period (daily or weekly), using Eq 15.

$$\Delta S=S_{f}-S_{i}$$ Eq 15

where Δ*S* is the soil water storage variation (mm), *S*_*f*_ the final soil water storage (mm), and *S*_*i*_ the initial soil water storage (mm).

Two phases of crop developments (intermediate phase and maturity phase) were selected for the comparison between ET derived from mass balance and PM method. The period of 5 weeks after planting was identified as the intermediate phase, which started from 10 July to 23 July, 2013. The maturity phase was when the canopy is fully developed starting from 14–27 August 2013.

## Results

### Meteorological and hydrological conditions

**Fig 2:**
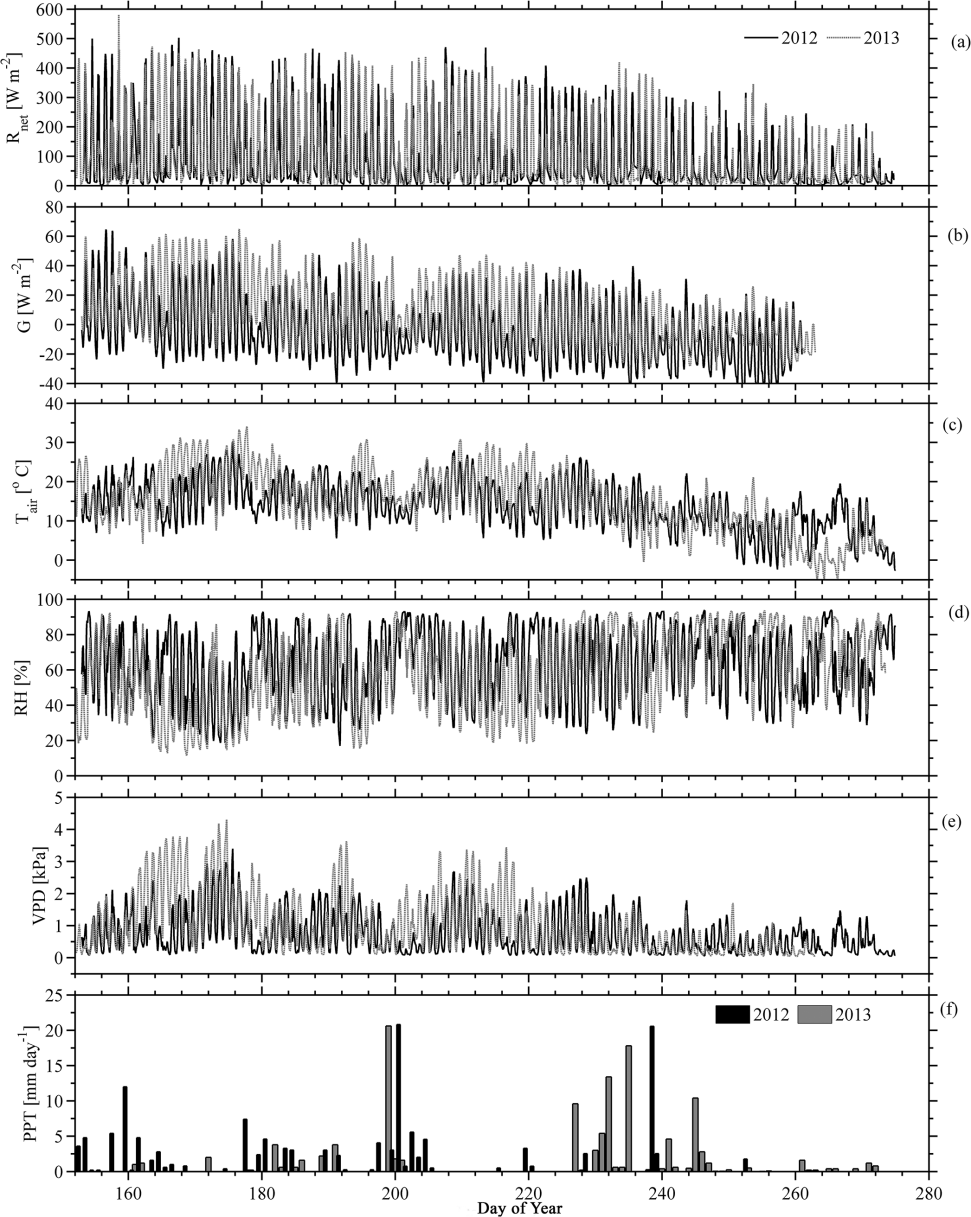
Half hourly meteorological time series during growing seasons in 2012 and 2013 at the experiment site.

**Fig 3:**
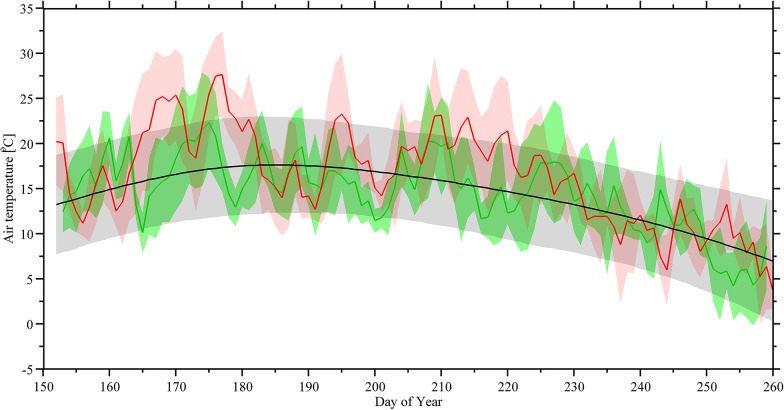
Time-series of average surface air temperature during growing season 2012 and 2013 compared with the thirty years climate data. The green line shows the daily mean of air temperature in 2012, the red line shows the daily mean of air temperature in 2013, and the black line shows the 30-year average of air temperature. Shading of each color provides an indication of the confidence range of the air temperature. The horizontal axis represents fractional Julian day in local AKST.

During the growing season, the average 30-min net radiation (*R*_*net*_) in summer 2013 was slightly higher than 2012 (Table 1), with the daytime average of 156±122 Wm^-2^ in 2013 and 149±123 Wm^-2^ (Fig 2A) in 2012. Conductive ground heat fluxes (G) at the site were calculated according to Eq 2 and found to be mostly proportional to *R*_*net*_ and following a diurnal cycle. On average, *G* in summer 2013 resulted to be similar to 2012 (Fig 2B). The mean air temperature was found to be on average 16.6°C in 2012 ranging from 0.2°C to 31.0°C and 18.2°C in 2013 with a variability range from -4.3°C and 34.9°C (Table 2). The growing season 2013 included ~5 days (44 hours) of negative air temperatures. However, the mean air temperatures in both years were higher than the 30-year average (Fig 3). The maximum air temperature reached to 31.0°C and 34.9°C during the month of June 2012 and 2013 respectively, while the normal (over 30 years) mean maximum was only 21.2°C (Table 2). This increase in maximum air temperature indicated a slightly warmer growing season in this high latitude agroecosystem. On the other hand, the minimum air temperature occurred in September with the lowest value of -4.3°C recorded in 2013.

**Table 1:** Seasonal means of major microclimate variables at FEF during the growing seasons under study.

Parameters	Growing season in 2012	Growing season in 2013
**R_net_**	149±123	156±122
**G_EC_**	8.0±5.8	7.5±5.6
**G_bare_**	21.1±18.8	13.1±8.7
**G_grass_**	18.2±13.7	8.1±5.7
**G_barley_**	20.0±16.9	21.5±18.8
**VPD**	0.6±0.5	1.15±0.92
**θ_ly_^a^**	0.3851±0.0174^b^	0.3685±0.0245 ^c^
**θ_unly_^a^**	-	0.2666±0.0659 ^d^
**θ_FEF_^a^**	0.1700±0.0197	-
**T_soil_ (°C)**	13.6±4.1	15.4±4.0
**U (m s^-1^)**	1.9±1.15	2.0±1.0

R_net:_ net radiation (W m^-2^), G_EC_: ground heat flux (W m^-2^) at EC site, bare field (G_bare_), brome grass field (G_grass_), and barley field (G_barley_), LE: latent heat flux (W m^-2^), VPD: vapor pressure deficit (kPa), θ_ly_: volumetric soil moisture content (m^3^ m^-3^) in irrigated vegetated lysimeter at 15 cm depth average from three lysimeters in summer 2012 and averaged from 0–20 cm depths from three lysimeters in summer 2013, θ_unly_: volumetric soil moisture content (m^3^ m^-3^) in unvegetated lysimeter in summer 2013, θ_FEF_: an average volumetric soil moisture content (m^3^ m^-3^) at 15 cm depth from brome grass, barley and bare field, T_s_: an average soil temperature (°C) at 15 cm from brome grass, barley and bare field, and U: wind speed (m s^-1^) at 2 m height at meteorological station. First column represents the major variables measured, second and third column are mean ± standard error of each variable for the 2012 and 2013 growing season.

^a^ More than two significant digits are needed for volumetric soil moisture content

^b^ volumetric soil moisture content data were available from June –27July, 2012

^c^ volumetric soil moisture content in a vegetated lysimeter for 2013 growing season

^d^ volumetric soil moisture content in an unvegetated lysimeter for 2013 growing season

**Table 2:** Monthly mean meteorological parameters measured at the FEF.

Month	Mean Temp. ± Std dev (°C)			Max Temp (°C)			Min Temp (°C)			Precipitation (mm)		
	Hist.	2012	2013	Hist.	2012	2013	Hist.	2012	2013	Hist.	2012	2013
**Jun**	15.8±5.4	17.6±4.9	22.7±6.3	22.0	31.4	34.9	9.6	4.9	3.8	34.8	53.0	4.4
**Jul**	16.9±5.1	17.0±4.2	19.1±4.8	22.6	28.2	32.0	11.3	6.3	7.8	54.9	53.6	36.6
**Aug**	13.2±5.3	14.9±4.6	15.9±5.9	18.8	26.8	31.1	8.0	5.7	0.3	47.7	30.5	56.2
**Sep**	6.4±6.7	5.6±3.2	5.9±5.2	12.6	12.8	21.3	1.7	0.2	-4.3	27.9	18.2	21.0
**Growing season** ^**c**^	14.4±2.9	16.6±4.9	18.2±6.4	21.2	24.8	29.8	7.6	4.3	1.9	165.3	155.3	118.2

Monthly means calculated between June to September during the 2012^a^ and 2013^a^ growing season in comparison with historical data of the climate normal^b^ in the 30-year time period from 1981–2010 for Fairbanks, Alaska, USA, provided by the National Climatic Data Center. Hist. represents the mean monthly historical climatological data in the 30-year period. The second-four columns indicate mean air temperatures with standard deviation (Std dev) during two summer seasons compared to the 30-year average.

^a^ Meteorological station at the study site

^b^The climate normal (a 30-year mean) at the Fairbanks International Airport (http://climate.gi.alaska.edu/Climate/Normals).

^C^ Here the growing period is calculated from 1 June to 20 September

The relative humidity (RH) of the experimental site averaged 69±19% for 2012 compared to 66±21% for 2013. High values of RH were correlated to lower air temperatures (Fig 2D) as well as to increased precipitation events. Half-hourly mean vapor pressure deficit (VPD) varied during the growing season as depicted in Fig 2E. The mean midday VPD was 0.6±0.5 and 1.2±0.9 kPa with the maximum VPD of 3.4 and 4.3 kPa in June 2012 and 2013, respectively (Table 1).

**Fig 4:**
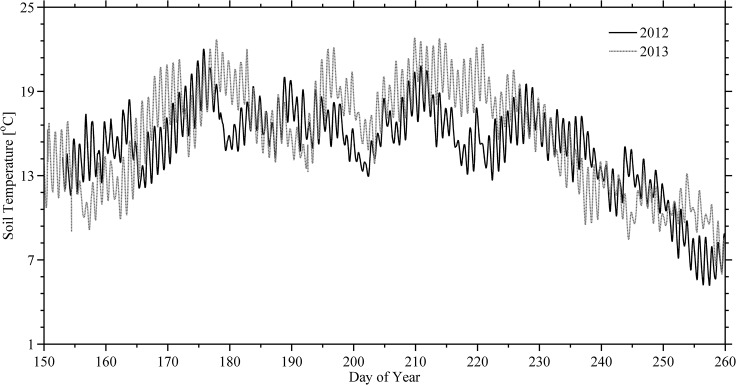
Time-series of soil temperatures. Soil temperature at 15 cm depth at the experiment site during 1 June to 17 September 2012 (black trace) and 2013 (gray trace). The horizontal axis represents fractional Julian day in local AKST.

The precipitation field was found to be very variable and significantly different from long term averages. Collected values at the experimental site for both years (Fig 2F) resulted in much lower amounts than those from 30-year average 165.3 mm (Table 2). On the other hand, comparing side-by-side both summers it was found that August 2013 (56.2 mm) verified larger amounts than August 2012 (30.5 mm) while the normal monthly average precipitation is (47.7 mm). Similarly, the driest period in the past 30 years was verified to be June of every year (mean precipitation of 34.8 mm); however, June 2013, showed in the study area, a precipitation of 4.4 mm that was below the 30-year average. In contrast, the amount of precipitation in June 2012 (53 mm) was higher than the normal average of June. In addition, precipitation decreased about 6% during the summer season of 2012 when compared to the long-term 30 years mean for this area [16]. A decreased rate of more than 28% was found in the summer 2013 resulting in abnormally dry conditions. In terms of T_soil_, there has not been significant differences in average T_soil_ measured at 15 cm depth at the experiment site when comparing the growing seasons of 2012 and 2013 (Fig 4; Table 1).

The soil volumetric water content (θ_ly_) in the lysimeters in the layer 0–15 cm (2012) and in the 0–20 cm depth (2013) varied greatly over the growing season. The variability of θ_ly_ depended on irrigation practice (i.e., irrigation quota and timing) and precipitation events. An average θ_ly_ was 0.3851±0.0174 (6 June to 27 July 2012) and 0.3685±0.0245 m^3^ m^-3^ (1 June to 6 September 2013) while an average θ_FEF_ in the farm field with no irrigation was 0.1700±0.0197 m^3^ m^-3^ average accounting for farm diversity of land surface type (e.g., crop land, grass land and bare land) (Table 1).

**Fig 5:**
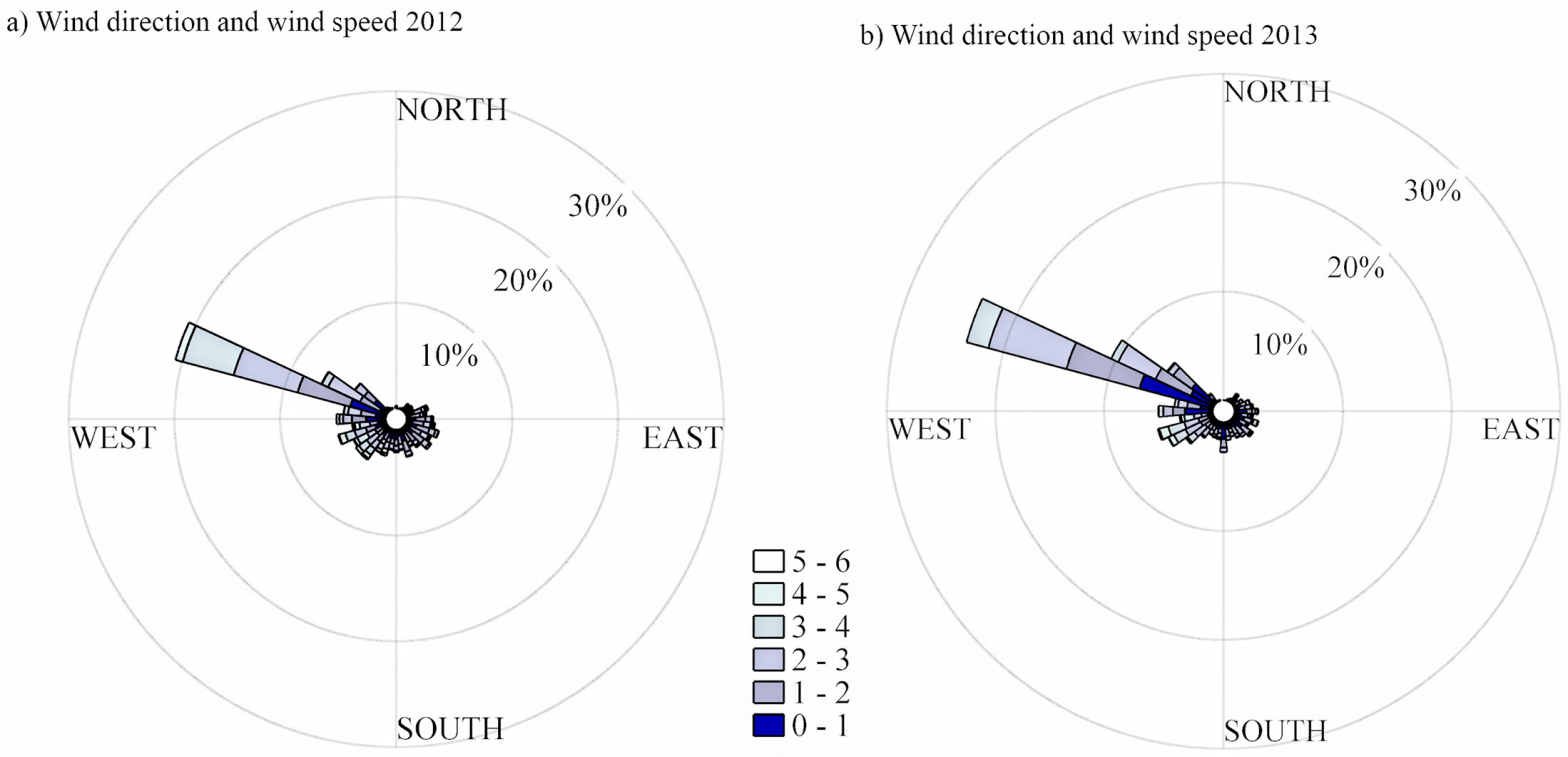
Frequency distribution of the wind speed and direction during summer. Left panel 2012 and right panel 2013 at the experiment site during the period of study at 2 m height.

The frequency distribution of surface wind direction and wind speed is shown during the period of study for the 2012 (Fig 5 panel on the left) and 2013 (Fig 5 panel on the rigth). Based on 30-min average, temporal series wind data illustrated that the prevalent wind direction was from northwest sector and varied from west-north-west to north-north-west and occurred about 30% and 36% of the time in 2012 and 2013, respectively. Additionally, the occurrence of surface winds from southwest was 22% for both years and from the southeast was 18% and 17% in 2012 and 2013, respectively. Wind speed, on the other hand, showed relatively steady values with an average value of 1.9±1.2 m s^-1^ and 2.0 ± 1.0 m s^-1^ with maximum at 6.7 m s^-1^ and 5.7 m s^-1^ in the 2012 and 2013 growing season, respectively.

In terms of the thermodynamic state of the surface atmospheric layer and soil conditions small differences were found in average air temperature, soil temperature, soil moisture, VPD, and wind speed during both years. However, in terms of preseason difference it has to be noted that the snow melt in 2013 extended to the 18 May while it reached only up to mid-March and melted on 22 April in 2012 [78–79].

### Surface Energy Balance

#### Energy closure at half-hour time-scale

**Fig 6:**
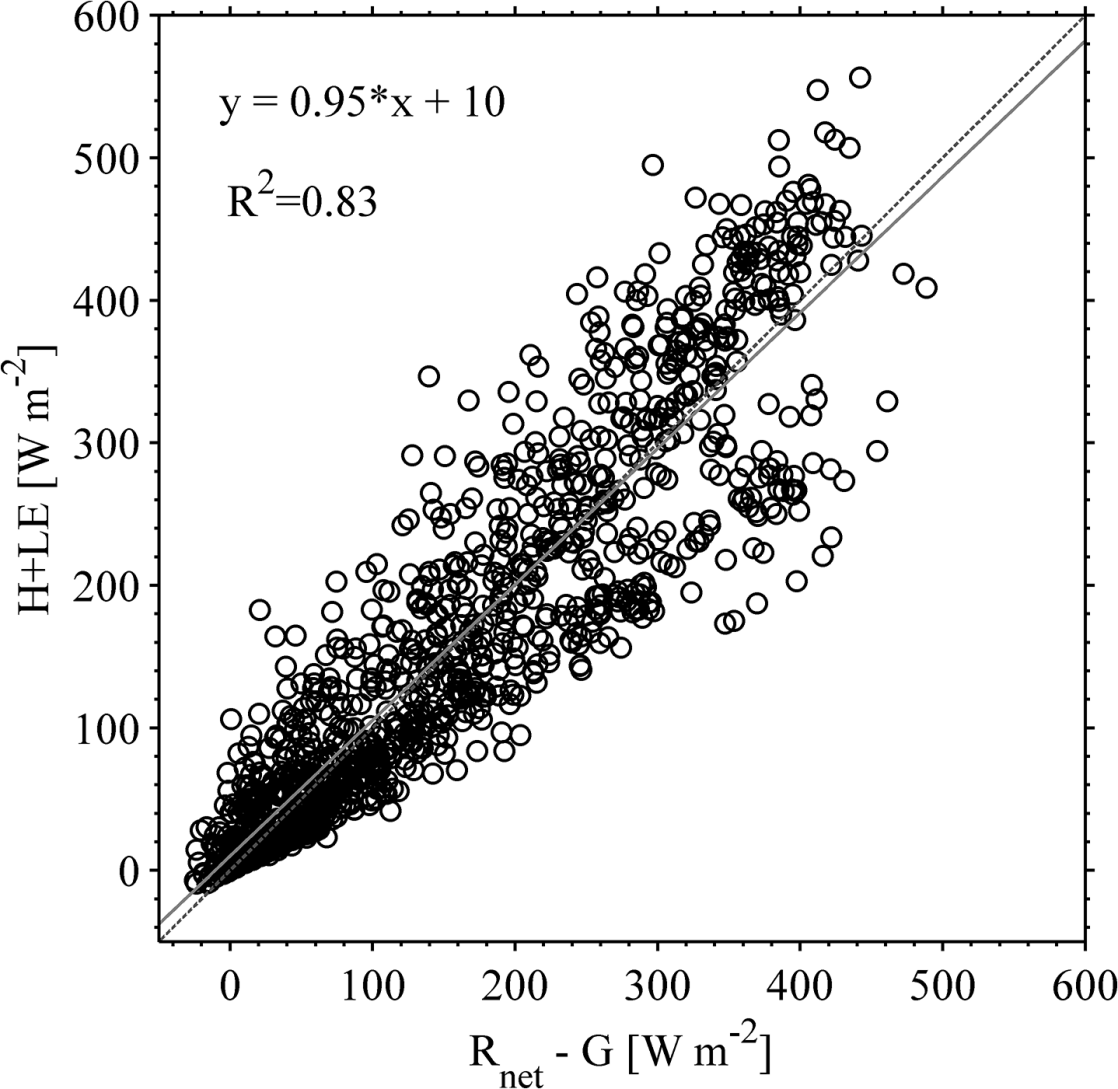
Scatterplot of energy balance closure. Horizontal axis is the available energy (*R*_*net*_ – *G*) for fluxes at the surface [W m^-2^] and vertical axis is the sum of turbulent fluxes of sensible (H) and latent heat (*LE*). Period of study (11 July to 31 August, summer 2013). Values were obtained are 30 minute averages under stationary conditions (1540 measurement points). Correlation coefficient was 95% with an offset of 10 W m^-2^.

The energy balance closure is defined as the ratio between the resulting turbulent fluxes manifested at the surface and the total energy available [80–82]. In this study, the energy balance closure was evaluated for the entire dataset 1,540 thirty-minute daytime intervals. There is a strong linear relationship between the sum of the 30 min average latent heat (***LE***) and sensible heat (***H***) plotted against the available energy (***R***_***net***_-***G***) for the summer 2013 growing season (Fig 6). A slope of 0.95 and an intercept of 10 W m^-2^ was obtained. These values indicate that on average the turbulent heat fluxes are slightly underestimated (by ~ 5%) neglecting the storage term in the energy balance equation due to the short canopy across the farm landscape (i.e., less than 0.50 m on average; see Fig 1). Similar results were found by Li et al. [83] over maize farmland in Northwestern region of China, while Parent and Actil [84] obtained 0.79 in a farmlands at Saint-Ubalde, South-Eastern Canada. Moreover in grasslands, energy balance closure values generally ranged from 0.70 to 0.80 [80], 0.70 in prairie [85], 0.74 in olive orchard field [86], 0.77 in switchgrass field [87], and 0.85 in an alfalfa field [88]. However in terrestrial ecosystems, particularly including forest [82, 89–93] the energy balance closure was found to range from 0.50 to 0.96 due to the complexities of the canopy architecture.

Therefore, it is concluded that the surface and atmospheric flow conditions obtained in this study (Fig 1) established the energy balance closure that is highly reliable and useful for examining energy partitioning among all energy fluxes.

#### Energy balance and energy partitioning

**Fig 7:**
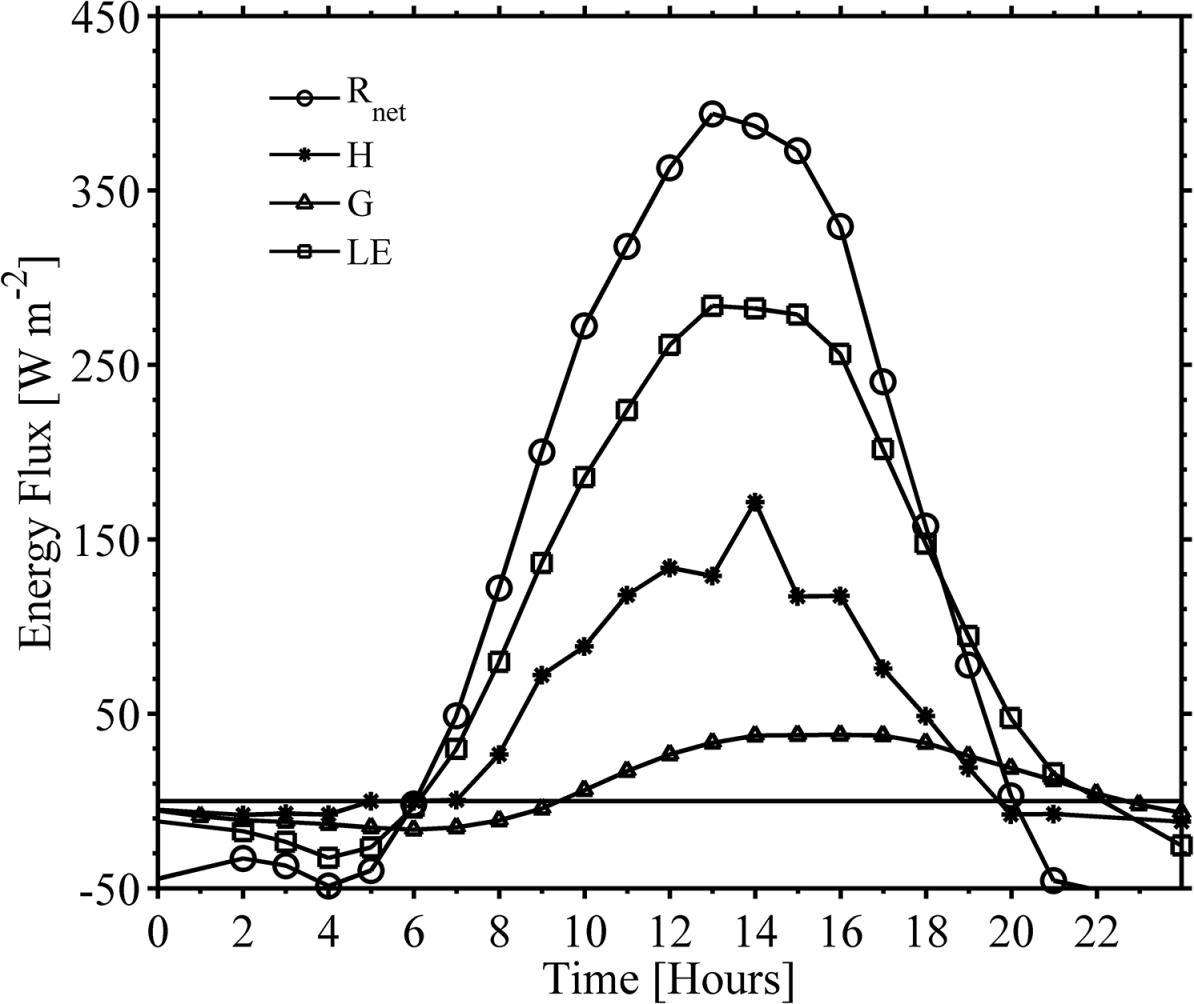
Diurnal cycle of radiative and turbulent fluxes during clear sky conditions. Case of 30 July (Day of Year 211) at the experiment site. Horizontal axis is in AKST time in [hrs.] and vertical axis is in W m^-2^. *R*_*net*_ = net radiation, *LE* = latent heat flux, *H* = sensible heat flux, *G* = ground heat flux.

An example of diurnal cycle of energy fluxes in the 2013 growing season is shown in Fig 7. In this case a clear sky day is shown in the central part of the growing stage (on 30 July, day of the year 211). The diurnal variation of ***LE*** flux was larger than the one for ***H*** flux. The values of ***LE*** gradually increased in the morning until it reached the peak value of 296 W m^-2^ around midday, basically following the time-variation of ***R***_***net***_. Then, ***LE*** rapidly decreased to zero at 2100 AKST when transition in the atmospheric surface layer started. On the same day, ***H*** flux slowly rose from 0 to a value of 180 W m^-2^, when ***R***_***net***_ peaked at 385 W m^-2^, then *H* declined steadily to zero at 1900 AKST indicating the changes in stability conditions in the atmospheric surface layer. The midday fraction of available energy (***R***_***net***_ – ***G***) into ***H*** was about 37%. On the same day, the ***G*** flux was the smallest compared with the rest of fluxes and became positive at 1000 AKST. The peak magnitude of ***G*** was 38 W m^-2^ and occurred at roughly 1600 AKST. Later on *G* dropped below zero about 2200 AKST ~two hours after ***R***_***net***_ turned negative. The calculated Bowen ratio (β) around middday was 0.6.

**Fig 8:**
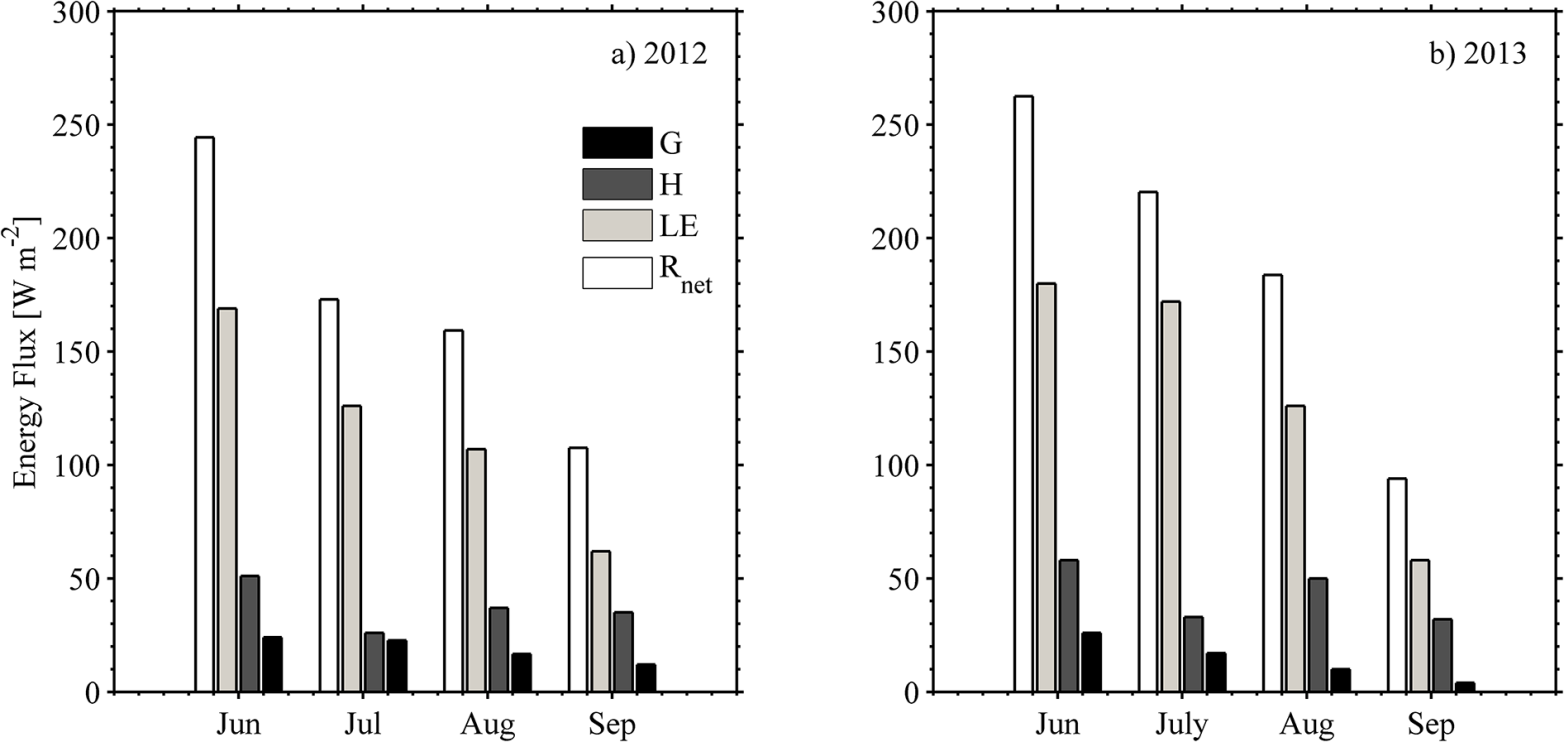
Monthly mean estimates for the four components of the surface energy balance (*R*_*net*_ = net radiation, *LE* = latent heat flux, *H* = sensible heat flux, *G* = ground heat flux). The series covers from June to September of 2012 (panel-a) and 2013 (panel-b).

The monthly midday averages of the energy balance components (*R*_*net*_, *LE*, *H*, *G*) for two years were calculated, and these results are illustrated in Fig 8. The data were acquired in the period June to September of each growing season. The summer mean *R*_*net*_ flux input for both years peaked in June (reaching ~ 244 W m^-2^ in 2012 and 264 W m^-2^ in 2013) and dropped-off gradually in August below 31–35% and September below 56–64% from the seasonal maximum (Fig 8). The amounts of *R*_*net*_ for both years were slightly different, with ~20 W m^-2^ in 2012 being lower than in 2013. When the midday means were considered over the period of the study, the mean *LE* was 119 W m^-2^ and 132 Wm^-2^, the mean *H* was 70 W m^-2^, 56 W m^-2^, and the mean *G* was 18.8 W m^-2^ and 16.7 W m^-2^, respectively, for summer 2012 and 2013. On average, *LE* in 2013 was about 17 W m^-2^ greater than in 2012; the maximum difference between both years was 57 W m^-2^ in July. In this subarctic farm, from June to September, the monthly mean of *LE* was greater than *H* and *G*, with a declining trend illustraed in July of each year. On the other hand, the maximum monthly mean of *H* occurred in July for both years and was approximately 80 W m^-2^, or about one half of the seasonal peak of *LE*. *G* is the smallest term in the energy balance equation. *G* was largest in June 2013 (number of times *G* was positive: 582 times from total of 698 times or ~ 83% positive) and July 2012 (number of *G* positive was 773 times from the total of 1193 times or ~ 65% positive) and slowly decreased after July until it appraoched around zero or negative number in September.

**Fig 9:**
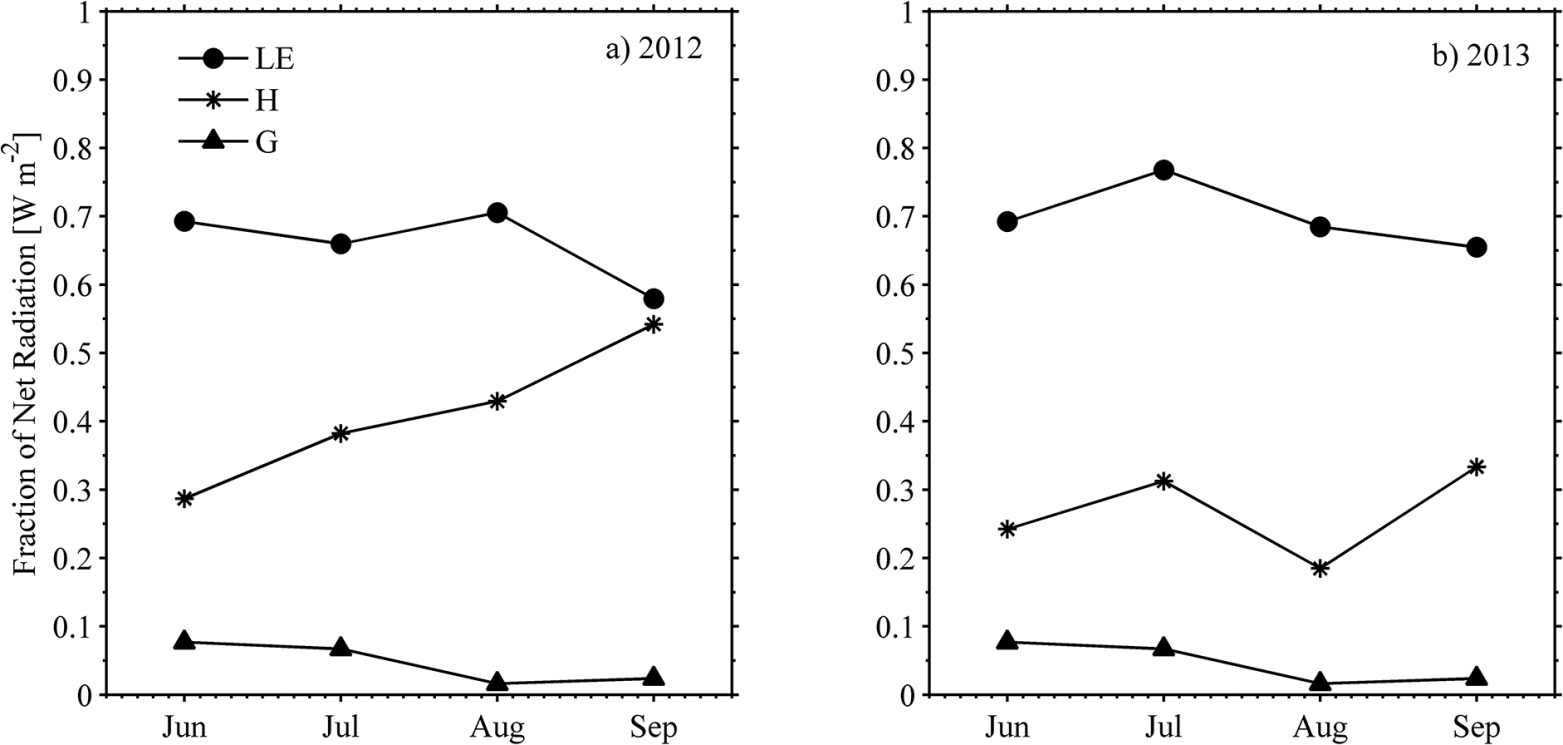
Monthly mean estimates of energy partitioning. *G*, *H* and *LE* are referred to *R*_*net*_. Panel-a represents 2012 and panel-b 2013 during the growing season. Statistical values to define the series are based on midday energy partitioning computed as a mean over 5 hours centered in solar noon.

Summertime energy balance partitioning was calculated based on the obtained experimental values. The mean values per variable during each year and the overall average for the entire two-years experiment are shown in Column 2, 3 and 4 respectively in Table 3. The fraction of the incoming *R*_*net*_ distributed across the variables in the energy balance components at the FEF during four months (June to September) of 2012 and 2013 growing seasons are shown in Fig 9. A similar pattern was observed in the time evolution of enegy fractions *LE*/*R*_*net*_ and *G*/*R*_*net*_. However, *H*/*R*_*net*_ during 2012 exhibited some divergence when compared with *LE*/*R*_*net*_. The values of *LE*/*R*_*net*_ ranged from 0.57 to 0.78 with an average of 0.67 for two growing seasons. The maximum of *LE* /*R*_*net*_ illustrated in June 2012 (Fig 9A) and July 2013 when the vegetation was fully developed (Fig 9A and 9B) slightly decreased after this month in both years following vegetation senescence as well as gradually decreased VPD in August 2012. The variations of VPD directly relate to *LE* and show a decreasing pattern after the greening period (June).

**Table 3:** Seasonal means of surface energy partitions, Bowen ratio (β), vapor pressure deficit (VPD), Priestley Taylor alpha coefficient (α) and energy balance closure (*C*_*F*_) at the FEF.

Parameters	Entire growing season 2012	Entire growing season 2013	Two years Average
***LE* /*R***_***net***_	0.63	0.67	0.67
***H* /*R***_***net***_	0.23	0.25	0.27
***G* /*R***_***net***_	0.11	0.07	0.06
**β**	0.36	0.38	0.40
**α**	1.03	0.77	0.91
***C***_***F***_	0.97	0.95	0.97

Values are calculated between 1 June to 15 September in 2012 and 2013 growing season. Average midday (1100–1500 AKST) energy balance energy partitioning obtained from a total 352 and 364 samples in 2012 and 2013, respectively. An average over the two-year period was calculated based on 716 data-points shown in the fourth column.

On the other hand, it was observed over the two years that the major portion of surface energy balance was attrributed to *LE* in the period June to September behaving similarly to that in tundra and wetlands in Arctic and Subarctic sites (Table 4). Nevertheless, *LE*/*R*_*net*_ value is comparable to the lower range variability of that obtained from maize and soybean farmland, Nebraska, USA (0.6 to 0.9) [94] and are close to the value of 0.68 obtained in commercial farms near Flora city, Florida USA [95]. In addition, the value of *H*/*R*_*net*_ ranged from 0.28 to 0.37 (mean 0.33) and 0.20 to 0.30 (mean 0.25) of *R*_*net*_ in 2012 and 2013, respectively. In comparison to other farmlands the ratio of *H*/*R*_*net*_ in this study was found slightly higher than in soybean and maize (0.2 to -0.2) according to experiments carried out in Nebraska [94] considering only periods of fully-developed canopies.

**Table 4:** Mean summer values of the energy balance partitioning for Arctic and Subarctic ecosystems calculated and/or collected from various published data sources.

Terrain type	Location	*LE* /*R*_*net*_	*H* /*R*_*net*_	*G* /*R*_*net*_	VPD	β	Methods	Source
**Agricultural land**	subarctic Interior Alaska	0.67	0.27	0.06	1.54	0.44	EC	(This work)^a^
**Wetland**	Schefferville, Quebec	0.63	0.25	0.10	-	0.50	EC	Moore et al. [128]
**Wetland**	Happy Valley, Alaska	0.57	0.29	0.09	-	0.50	EC	Harazono et al. [120]
**Wetland**	Churchill, Manitoba	0.65	0.20	0.11	1.06	0.31	BREB	Rouse [121]
**Arctic coastal wetland**	Barrow Alaska	0.28	0.35	0.15	0.12	1.25	EC	Liljedahl et al. [99]
**Moist tussock Tundra**	Happy Valley Alaska	0.43	0.37	0.14	-	0.9	EC	Vourlitis and Oechel [89]
**Moist Tussock Tundra**	Seward Peninsula, Alaska	0.36	0.34	0.12	0.52	0.94	EC	Beringer et al. [122]
**Upland Tundra**	Hudson Bay Coast, Ontario	0.57	0.29	0.09	-	0.51	BREB	Rouse et al. [129]
**Upland Tundra**	Happy Valley, Alaska	0.49	0.40	0.16	0.81	0.82	EC	Harazono et al. [120]
**Upland Tundra**	Ice Cut, Alaska	0.61	0.27	0.12	-	0.44	EC	Eugster et al. [44]
**Tundra (non-shrub wet fen)**	Imnavait Creek, Alaska	0.67	0.26	0.07	-	0.39	EC	Eugster et al. [44]
**Tree line shrub tundra**	Wiseman, Alaska	0.65	0.30	0.05	-	0.46	EC	Eugster et al. [44]
**Black Spruce forest**	UAF	0.20	0.39	0.03	-	2.03	EC	Starkenburg et al. [96]
**White spruce forest**	Seward Peninsula, Alaska	0.37	0.44	0.05	0.39	1.22	EC	Beringer et al. [122]
**Black spruce forest**	Delta Junction, Interior Alaska	0.24	0.58	0.03	-	2.42	EC	Lui et al.[130]
**Black spruce forest**	Poker Flat, Interior Alaska	0.37	0.35	0.26	0.5	0.95	EC	Nakai et al.[123]

First column represents the ecosystem types, second column is the location of measuring site, third to fifth columns are energy partitioning values for *LE* /*R*_*net*_, *H* /*R*_*net*_, *G* /*R*_*net*_ (derived from daily midday flux averages), sixth column is VPD (kPa) for each ecosystem type, seventh column is the Bowen ratio (β), eigth column is the measuring method used for energy budget components measured, and nineth column is the reference for data. EC = Eddy covariance method. BREB = Bowen ratio-energy balance method.

^a^Average over two years growing season data in 2012 and 2013 from this present work during 1 June—20 September.

At all instances the β in the experiment site during the summer 2012 and 2013 was found to be systematically less than unity and ranging from 0.3 to 0.9 with an average of 0.36 in 2012 and 0.38 in 2013 (Table 3). On the other hand, *G*/*R*_*net*_ ranged from 0.05 to 0.11 in 2012 (mean 0.06) and 0.01 to 0.08 (mean 0.07) in 2013 of *R*_*net*_. Large values of *G* are typically found in subarctic landscapes [95] with the exception of boreal forests [96]. Nevertheless, the ratios of *H*/*R*_*net*_ and *G*/*R*_*net*_ were in the interval of observed values from wetlands and shrub tundra (*H*/*R*_*net*_ near 28%, *G*/*R*_*net*_ ranges from 6–12%) from the Western and Central Canadian Subarctic [97].

In terms of evaluating the general trends on ET associated with changes in vegetation, soil moisture, and meteorological parameters [69] the Priestley-Taylor coefficient (α) was calculated. High values of α are associated with a high-energy partition of *LE*/*R*_*net*_, while low value represents the opposite. Stewart and Rouse [98] found that the theoretical value of α = 1.26 is generally applied to saturated surfaces in high latitude. However, in the present study α = 0.91 was the average from two years while α ranging from 0.40 to 1.22 is reported for Arctic and boreal ecosystems [44]. The average value of 0.91 in this study is consistent with values reported by Eaton et al. [97] in upland tundra. This variable range of α depends on the specific ecosystems under consideration for example, Liljedahl et al. [99] reported mean midday α = 1.08 (offshore) and 0.95 (onshore) in Arctic coastal wetland.

### Evapotranspiration from water balance equation

#### ET by mass balance from irrigated lysimeters versus ET by energy balance

ET based on energy balance was obtained using Penman-Monteith (ET_PM_) approach. ET_PM_ was computed based on available meteorological variables collected at the site (Fig 2), **Eq 4**according to Monteith [100]. The cumulative ET from mass balance obtained by **Eq 12**was applied to the irrigated vegetated (ET_VL_) and unvegetated (ET_UVL_) lysimeters. The measurements of precipitation (***P***), irrigation (***I***), drainage (***D***), and change in storage (*Δ* ***S***) allowed estimating ET.

Comparing ET rates among different treatments on lysimeters, the results showed ET_VL_ having from 5 to 25% larger cumulative ET compared to ET_UVL_. When considering ET_PM_ as reference of a larger evaporative area, the ratios ET_VL_/ ET_PM_ and ET_UVL_/ ET_PM_ verified a lower fraction than 1 during the first week. While, for the rest of the experiment, it resulted in a ratio larger than or fairly close to 1 (Table 5). Similar results were obtained by Braley [48] based upon their study within irrigated lysimeter and non-irrigated lysimeter in 1979. On the other hand, the ratio of ET_VL_/ ET_PM_ was found to be slightly higher than ET_UVL_/ ET_PM_ (Table 5). Nevertheless, on average ET mass balance was mostly higher than ET energy balance due to additional water input from irrigation. The average ratios of ET_VL_/ ET_PM_ and ET_UVL_/ ET_PM_ were found to be 1.12 and 0.97_,_ respectively. However, the ratio of ET from mass balance to the measurement of pan evaporation averaged 0.59 and 0.66 for ET_UVL_ and ET_VL,_ respectively.

**Table 5:** A summary of weekly ET by water balance in comparison with the ET by energy balance during intermediate development phase (10–23 July, 2013) and maturity phase (14–27 August, 2013) of crop under wet conditions.

Week-period	ET_VL_ (mm)	ET_UVL_ (mm)	ET_PM_ (mm)	ET_VL_/ ET_PM_	ET_UVL_/ ET_PM_
**10–16 July, 2013**	22.91	21.57	25.81	0.89	0.84
**17–23 July, 2013**	22.26	20.64	20.54	1.08	1.00
**14–20 August, 2013**	20.86	15.59	15.00	1.39	1.04
**21–27 August, 2013**	18.50	16.47	16.47	1.12	1.00

First column is time period covered by measurement (i.e., 10–16 July, 2013), second column is a weekly accumulated ET in the vegetated lysimeter (ET_VL_), third column is a weekly accumulated ET in unvegetated lysimeter (ET_UVL_), fourth column is a weekly accumulated ET by energy balance (ET_PM_) derived using the Penman Monteith equation, fifth column is the ratio of ET_VL_/ ET_PM_, and sixth column is the ratio of ET_UVL_ / ET_PM_.

Additionally, the ET estimate from water mass balance approach provided higher rates than from energy balance approach, and this difference was accentuated as the vegetation fully developed (Table 5). However, ET from energy balance method can be used as a reference ET for an agroecosystem especially in the sparse vegetation landscape.

In order to compare and benchmark the hydrological rates in agricultural lands, Table 6 shows the annual and summer hydrological balance characteristics among various ecosystems in Arctic and Subarctic regions. Based on, the total precipitation of 65 mm and irrigation 41.2 mm during this study period, the ET_VL_ was almost 97% while the ET_UVL_ was approximately 88% of precipitation and irrigation. In contrast, lower percentages indicated in Imnavait Creek Basin in North Slope of Alaska reported that 50% of precipitation went through the ET process and only 36% was found in the Upper Kuparuk Alaskan watershed [101] In addition, 76% of precipitation was found to be evaporated from the permafrost in the boreal forest at Caribou-Poker Creek Watershed in Interior Alaska [102]. Nevertheless, other studies have also shown a lower ratio of precipitation being evaporated through ET process when compared to this present study [103–109] (Table 6).

**Table 6:** The annual and summer hydrological balance characteristics for Arctic and Sub-arctic regions compiled from various published data sources.

	Location/latitude, longitude	P	Runoff	ET	ΔS	ET/P	Source
**1**	VL at the UAF AFES FEF, Alaska, USA (64. 5°N 147. 5° W)	106.2^a^	0	103.18	-0.07	0.97	[This work]^b^
**2**	UVL at the UAF AFES FEF, Alaska, USA (64. 5°N 147. 5° W)	106.2^a^	0	92.99	-0.93	0.87	[This work]^b^
**3**	Imnavait Creek (kuparuk), Alaska, USA (68. 6°N 149. 4° W)	359	181	179	-	0.50	Kane et al. [71]
**4**	Upper Kuparuk, Alaska, USA (68. 6°N 149. 4° W)	376	237	140	-	0.37	Kane et al. [100]
**5**	C2, Caribou-Poker Creek, Alaska,USA (65. 2°N 147. 5° W)	412	80	312	15	0.75	Bolton et al. [101]
**6**	Tiksi, Russia (71. 7°N 128. 8° W)	98	144	54	-17	0.55	Ishii et al. [102] ^b^
**7**	Havikpak Creek, Canada (68. 3°N 133. 5° W)	283	110	134	-	0.47	Marsh et al. [103]
**8**	Scotty Creek, Canada (61. 3°N 121. 3° W)	421	148	282	-	0.68	Quinton et al. [104]
**9**	Dead Creek, Canada (50. 0°N 95. 0° W)	526	103	423	-	0.80	Thorne and Hawkins [105]
**10**	Iittovuoma, Finland (68. 8°N 25. 4° E)	573	342	231	-	0.40	Seuna and Linjama [106]
**11**	Filiper River (Mogot), Russia (56. 6°N 124. 9° E)	319	168	169	-8	0.53	Vailenko [107]^b^
**12**	Kontakovy Creek, Russia (68.7. 3°N 133. 5° W)	405	296	137	0	0.34	Zhuravin [108]
**13**	Trail Valley Creek, Canada (68.7. 3°N 133. 5° W)	231	118	110	4	0.48	Zhuravin [109]

^a^ Precipitation+Irrigation

^b^ Summer hydrological balance

#### Penman- Monteith Evapotranspiration (ETPM) and Pan Evaporation (EP)

**Fig 10:**
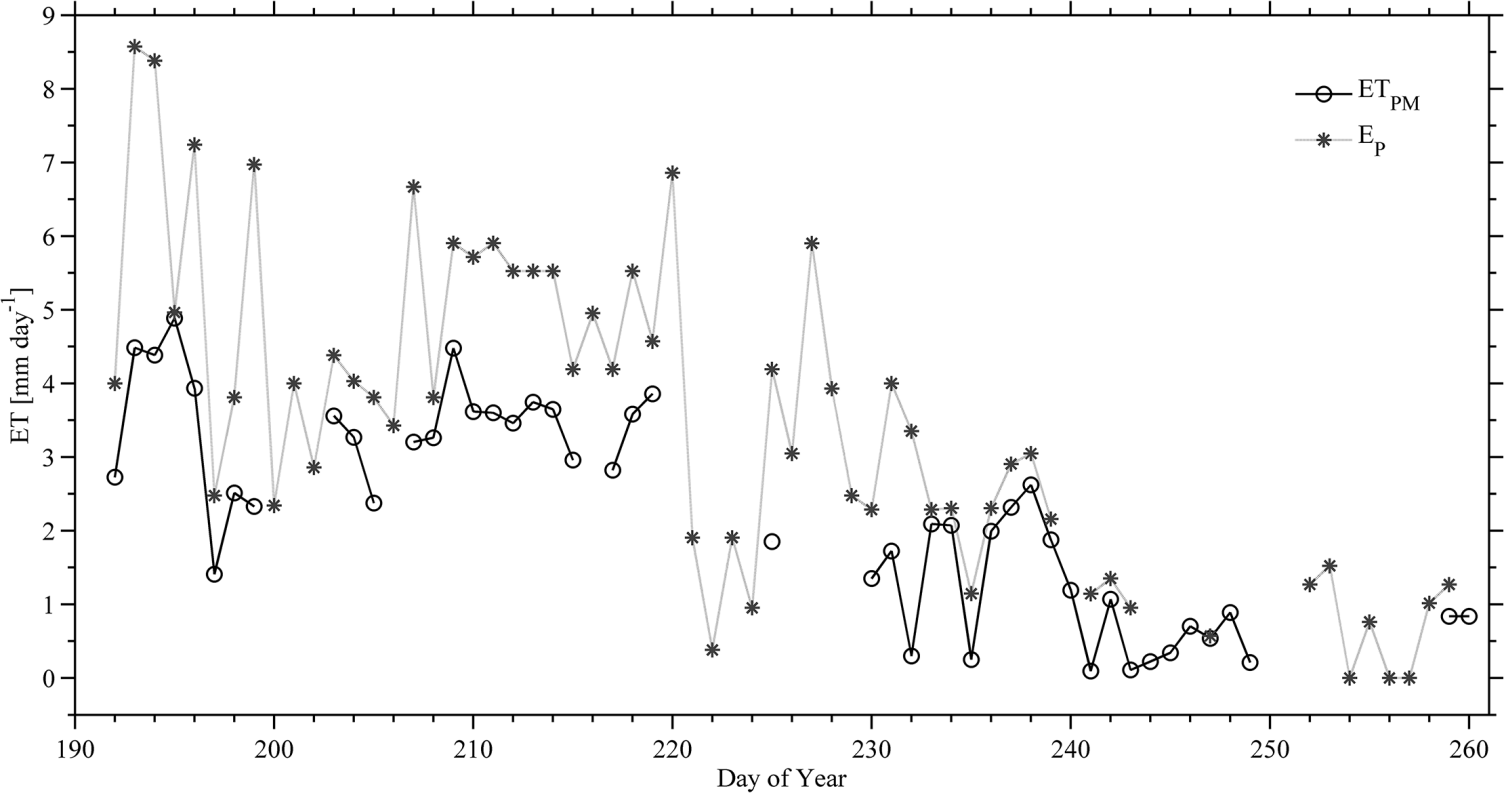
Daily means of evapotranspiration. Measured by pan evaporation (E_P_) and estimated based on Penman Monteith (ET_PM_) from 10 July to 16 September 2013. Some data gaps were caused by power interruptions and instrument failure and repair.

Potential ET was measured with a Class A evaporation pan (E_P_). Daily pan evaporation determinations (E_P_) were manually made at 0800 AKST and no later than 0815 AKST every day. The E_P_ fraction is defined as the potential evaporation rate for a given location. E_P_ in this study ranged from 0 to more than 8.57 mm per day under clear skies conditions with daily average of 3.44±2.15 mm per day. Because manual E_P_ measurements were made at one given time everyday the temporal series of ET_PM_ was then compiled for a similar time interval for daily estimates comparison. There were about 89 measured values of E_P_ available and only 69 values were used for comparison with ET_PM_ because of sensor malfunctioning (S1 Table). On the other hand, during the study period, relatively high rates of ET_PM_ were recorded in the month of July, while a declining trend was shown in September (Fig 10). Daily values of ET_PM_ ranged from less than 1 mm to more than 4 mm and the daily average was 2.27±1.40 mm per day. This average value is slightly higher than values obtained by Braley [48] for the same site. The results showed that overall daily values of E_P_ exceeded ET_PM_. Regression analysis was used to relate ET_PM_ to E_P_ and a correlation (R^2^ = 0.69) was found, while a poor correlation (R^2^ = 0.38) between those values was documented in other environments Florida, USA [95].

## Discussion

This study investigated the energy and water mass balance during 2012 and 2013 growing season at the UAF AFES FEF representative of high latitude agricultural lands in Interior Alaska. The summertime climatic characteristics at the site during both years were examined. An increase in the mean air temperature of +2.2°C and +3.7°C was observed in 2012 and 2013, respectively, when compared to the 30-year average of mean air temperature. Nevertheless, the mean air temperature regime during 2012 resulted within the normal range of variability for 30-year climatological data (Table 2). It is worth mentioning, that the mean values during 2013 verified a temperature excursion larger than one standard deviation when compared with the 30-year climatological mean. This warmer mean air temperature observed in summer 2013 is consistent with recent results indicating an increase in air temperatures of 1.4°C for Interior Alaska during the last 100-year record [16]. On the other hand, summer precipitation for 2012 remained approximately within the range of the 30-year average (165.35 mm); while, precipitation for 2013 was ~26 mm below the normal average. After statistical examination of time series of *R*_*net*_ values it was concluded that no major differences between years were found (Table 1 and Fig 8).

In terms of turbulent flux regimes, the sensible heat verified no major variations with an average of ~ 80 W m^-2^. However latent heat fluxes increased by ~ 22 W m^-2^ during summer 2013. This slight positive trend could only be explained by the change in pre-season conditions that made the sub surface to be wetter through an extended snowmelt period than in the other year (2012). [79–80].

The value of energy balance closure C_F_ found in the field experiment reached levels of ~0.95 (Fig 6). This value is considered to be representative of energy closure in agricultural fields because often the topography characterizing such systems is close to ideal conditions (i.e., flat terrain covered by short grass). Similarly, we found this value to be in good agreement to closure levels 0.70 to 0.99 observed at Fluxnet sites including several agricultural lands [91]. Still, a small residual term was found ~3%. This term is generally attributed to systematic methodological errors, systematic instrument bias, neglected energy sinks, and unrepresentativeness of the *G* term [81, 91, 93, 109–113]. After a careful revision of all terms intervening in the energy balance the residual term, can only arise from surface patches containing different crops (e.g., bare land, grassland and trees). Therefore, an evaluation of *G* was conducted over the mentioned surface patches and it was observed to vary 3–6% (Table 1). This attribution is in agreement with other reports [93, 114] in which *G* was found to dominate the relative uncertainty on the surface energy balance closure reaching up to 20% in agriculture sites [115–116].

The energy partitioning of *R*_*net*_ into *H*, *LE* and *G* is strongly influenced by changes in surface conditions such as dynamics of vegetation growth, changes in soil moisture and surface temperature affected by precipitation [117–119]. In particular the relationship between *LE* and soil moisture is complex, variable in space and time and verifies nonlinear relationships with the energy balance terms. For example, *LE*/*R*_*net*_ was found practically similar in dry and wet soils constrained in the case by VPD<0.30 kPa in Arctic coastal wetland [99].

In the present study, the energy ratio of *LE/R*_*net*_ was found to be systematically larger than the ratio for *H/R*_*net*_ and *G/R*_*net*_ consistently also with β < 1 (Table 3). Likewise, several ecosystems in the Arctic and Subarctic have larger *LE* /*R*_*net*_ than *H*/*R*_*net*_ [44, 45, 97, 120–124] (Table 4). Alternatively, we have found that in comparison to some Arctic ecosystems [44, 97, 99, 125–126] this ratio *LE* /*R*_*net*_ is largest amongst the other fractions. However, we have to point out that this ratio is still on the lower range interval when compared to mid-latitude agricultural fields [127].

The monthly trends of energy fractions accounting for their seasonal evolution were observed to maximize around the middle of the summer to then trend negatively to the end of the season (Fig 9). This behavior is verified in the case of *G*/*R*_*net*_ and *LE*/*R*_*net*_. However, the energy ratio *H*/*R*_*net*_ is observed to fluctuate at the end of the season in 2012. These variations in *H*/*R*_*net*_ are consistent to changes in the thermodynamics of the air mass as indicated in Fig 4. Similarly the energy fraction associated to *LE* /*R*_*net*_ verifies a positive trend during the decaying phase of the season demonstrating an increasing response to monthly precipitation during August 2012 (Table 2).

With the aim to identify agricultural land energy fractions in the framework of natural ecosystems in high latitudes, Table 4, reports a comprehensive comparison among these systems across the panArctic. For Arctic and subarctic wetlands, *LE*/*R*_*net*_ was reported to be larger than 0.57 according to the studies of Moore et al.[128], Harazono et al. [119], and Rouse [121]. In contrast, a lower value of *LE*/*R*_*net*_ in Arctic coastal wetlands was observed. In this case, a different environmental forcing due to the presence of onshore wind constantly offsets the energy partitioning [99]. While on the other hand, the reported values in literature of *H*/*R*_*net*_ and *G*/*R*_*net*_ are similar to the ones in the present study.

Furthermore the energy partitioning in tundra ecosystem verifies in comparison mostly a lower *LE*/*R*_*net*_ ranging from 0.36 to 0.67 [44, 91, 120, 122, 129]. On the other hand, *H*/*R*_*net*_ in the present study compares well with the lower range reported from the mentioned studies in the range 0.26 to 0.40. Finally the energy fractions obtained in this study correspond well with the results obtained in upland tundra ecosystems reported by Eugster et al. [44] in which *LE* was the dominant component of surface energy balance. Conversely, energy balance studies in Alaskan coniferous boreal forest (i.e, composed mainly of white and black spruce trees) have found that *H* dominated the energy balance [96, 122, 130) with the exception of the study of Nakai et al. [123] in the Poker Flat Research Range which indicated *LE* slightly dominant on a sparser canopy over discontinuous permafrost.

In terms of evaluating the ET rates, this study produced two different approaches based on mass balance (i.e., lysimeters based) and energy balance (i.e., micrometeorological based). These two approaches have definitively different spatial and temporal scales in terms of their environmental interactions [131] and therefore their ET rates were slightly different owing to the vegetation development and the spatial scale representation. In order to evaluate the potential for environmental interaction of agricultural land the lysimeter experiment was conducted based on irrigation practices over two treatments: vegetated and unvegetated. Overall this study found that ET_VL_ was higher than ET_UVL_ while ET_UVL_ was similar across the season to ET_PM_ (Table 5). However, it is important to note that if ET_PM_ is taken as the reference, the ratios ET_VL_/ET_PM_ and ET_UVL_/ET_PM_ verified a lower fraction in the first week due to the development phase of the vegetation. On the other hand, the fraction ET_UVL_/ET_VL_ represented the percentage of ET due to vegetation growth and interception ranging from 75 to 94% (Table 5).

To give a prospective impact of agricultural lands in the framework of high latitude environments, Table 6 brings similar data records around the pan-Arctic from ten sites which are compared to the findings of this study. Establishing the ratio ET/P allows the evaluation of the percentage of precipitation input that is sent back to the atmosphere through ET. Based on the synthesis of Arctic basins hydrology study by Kane and Yang the ET/P ratio is well-correlated to latitude in Arctic natural ecosystems and basically accounts for 36–75% of the mass balance (Table 6) [101–109]. Whereas, in this study this fraction ranged much higher from 87% to 97%; only comparable to the ones obtained by Thorne and Hawkins [106] calculating 80% return (Table 6). These differences in ET and ET/P ratios are due to availability of energy for fluxes at the surface [132–133], precipitation distribution and rate as well as topography [134], forest canopy interception capacity associated to tree species and leaf area index [135]. It is therefore concluded here that the fraction of ET returned to the atmosphere in agricultural lands represent a much larger fraction of what has been reported for boreal forest at approximately the same latitude [96] and also larger than the fractions obtained in Arctic tundra.

## Conclusions

We found that the ET cycles represent a large portion of surface energy balance partitioning accounting for approximately 67% of the net radiation. The ratio of ET obtained by water mass balance related to the measured potential ET ranged from 0.59 to 0.66 for evapotranspiration rates based on unvegetated and vegetated lysimeters, respectively. Additionally, ET was responsible for removing 97% and 88% of the moisture added to the vegetated and non-vegetated lysimeters, respectively.

Northern latitudes are characterized by diverse ecosystems where wetlands and tundra dominate Arctic regions, and boreal forest with coniferous and deciduous trees dominate Subarctic regions. This work puts in perspective and compares the surface energy fraction on agricultural lands in the context of boreal forests, Arctic wetlands and tundra (Tables 4 and 6). The results indicate that the energy fluxing regime in terms of ET/*R*_*net*_ of agroecosystems in the subarctic exhibits similar characteristics to tundra in the Arctic; contrasting therefore with subarctic boreal forest. Moreover, differential fluxes may manifest between agricultural and boreal forest over short spatial scales forcing small-scale circulations creating an additional imbalance term in the energy budget. Therefore, this study indicates that the presence and further development of agroecosystems in northern latitudes may lead to significant changes of ET cycle during the growing season in comparison with natural existing ecosystems.

Consequently, replacing native ecosystems to promote agricultural development and economic activities may result in significant changes in the land-use and therefore in surface energy regimes and balance. Moreover, these changes can collectivelly upscale to shift seasonal magnitude (season length and timing) and temporal partitioning of regional fluxes introducing a positive feedbak to climate.

Finally, on the basis of a changing climate scenario manifested through increasing air temperatures, lengthening of growing season and changes in vegetation gradients in northern latitudes, expanding agricultural lands may lead to an increase of ET cycles (water vapor return to the atmosphere) that will propagate to larger atmospheric scales through nonlinear interactions characterizing the surface-atmosphere system.

## Supporting Information

S1 Fig Ensemble instrumentation during intensive observation periods. (DOCX)

S1 Table Instrumentation utilized in the UAF AFES Fairbanks Experiment Farm in summers of 2012 and 2013. (DOCX)

S2 Table Additional Instrumentation. (DOCX)
