# Supplementary material for: Evapotranspiration Cycles in a High Latitude Agroecosystem: Potential Warming Role
Source: PLoS One. 2015 Sep 14;10(9):e0137209. doi: 10.1371/journal.pone.0137209 (PMC4569083; doi:10.1371/journal.pone.0137209)
Supplement: S1 Fig — (DOCX) [file pone.0137209.s001.docx]

S1. Ensemble instrumentation during intensive observation observing measurement periods

| (a)   | (b)   |
| --- | --- |

**Fig A.** Panel (a) – (b) represents the lysimeter setup including drainage system and installation probes layout for summer 2012 and 2013.
