# Supplementary material for: Evapotranspiration Cycles in a High Latitude Agroecosystem: Potential Warming Role
Source: PLoS One. 2015 Sep 14;10(9):e0137209. doi: 10.1371/journal.pone.0137209 (PMC4569083; doi:10.1371/journal.pone.0137209)
Supplement: S1 Table — (DOCX) [file pone.0137209.s002.docx]

**S1.** Instrumentation utilized in the UAF AFES Fairbanks Experiment Farm in summers of 2012 and 2013.

| **Instruments**  **& symbol** | **Growing season - 2012** | **Growing season - 2013** |
| --- | --- | --- |

|  | **Jun** | | | | **Jul** | | | | **Aug** | | | | **Sep** | | | | **Jun** | | | | **Jul** | | | | **Aug** | | | | **Sep** | | | |
| --- | --- | --- | --- | --- | --- | --- | --- | --- | --- | --- | --- | --- | --- | --- | --- | --- | --- | --- | --- | --- | --- | --- | --- | --- | --- | --- | --- | --- | --- | --- | --- | --- |
|  |  | | | |  | | | |  | | | |  | | | |  | | | |  | | | |  | | | |  | | | |
| **T_air_** | - | - | - | - | - | - | - | - | - | - | - | - | - |  | - | - | - | - | - | - | - | - | - | - | - | - | - | - | - |  | - | - |
| **RH** | - | - | - | - | - | - | - | - | - | - | - | - | - |  | - | - | - | - | - | - | - | - | - | - | - | - | - | - | - |  | - | - |
| **P** | - | - | - | - | - | - | - | - | - | - | - | - | - |  | - | - | - | - | - | - | - | - | - | - | - | - | - | - | - |  | - | - |
| **θ_ly,unly_** |  | - | - | - | - | - | - |  |  |  |  |  | - | - |  |  |  | - | - | - | - | - | - | - | - | - | - | - | - |  |  |  |
| **θ_FEF_** | - | - | - | - | - | - | - | - | - | - | - | - | - | - | - | - |  |  |  |  |  |  |  |  |  |  |  |  |  |  |  |  |
| **T_soil_** | - | - | - | - | - | - | - | - | - | - | - | - | - | - | - | - | - | - | - | - | - | - | - | - | - | - | - | - | - | - | - | - |
| **u_i_,T** |  |  |  |  |  |  |  |  |  |  |  |  |  |  |  |  |  |  |  |  |  | - |  | - | - | - | - | - | - | - |  |  |
| **R_net_** | - | - | - | - | - | - | - | - | - | - | - | - | - | - | - | - | - | - | - | - | - | - | - | - | - | - | - | - | - | - | - | - |
| **LAS** |  |  |  |  |  | - | - |  |  | - | - |  |  |  |  |  |  |  |  |  |  | - |  | - | - | - | - | - | - | - | - |  |
| **PE** |  |  |  |  |  |  |  |  |  |  |  |  |  |  |  |  |  |  | - | - | - | - | - | - | - | - | - | - | - | - |  |  |
|  |  |  |  |  |  |  |  |  |  |  |  |  |  |  |  |  |  |  |  |  |  |  |  |  |  |  |  |  |  |  |  |  |

The observations were made during the growing season experiment (June-September). Timeline for involved instruments is indicated by shaded gray color in the table. Sensor description: T_air_ (ambient temperature sensors), RH (relative humidity probes 2 m and 5m), P (ambient pressure) probes, θ_ly_ and θ_unly_ (volumetric soil moisture sensor in lysimeter plot), θ_FEF_ (volumetric soil moisture sensor under barley, brome grass and bare field at FEF), T_soil_ (soil temperature sensors), sonic anemometer (u_i_ is u, v, w and T is sonic temperature), R_net_ (net radiation sensor), LAS (large aperture scintillometer) and PE (class A pan evaporation).
