# Supplementary material for: Evapotranspiration Cycles in a High Latitude Agroecosystem: Potential Warming Role
Source: PLoS One. 2015 Sep 14;10(9):e0137209. doi: 10.1371/journal.pone.0137209 (PMC4569083; doi:10.1371/journal.pone.0137209)
Supplement: S2 Table — (DOCX) [file pone.0137209.s003.docx]

S2. Additional Instrumentation

| **Instrument & Symbol** | **Model** | **Variable Measured** | **Mounting Height [m]** |
| --- | --- | --- | --- |
| **u _,_T** | RMYoung 81000 | u, v, w and sonic temperature | 3.5 |
| **RH** | Film capacitor element | relative humidity | 2, 5 |
| **R_net_** | NRLite Campbell Scientific | solar radiation | 1 |
| **T_air_** | PT 107 Campbell Scientific | air temperature | 1, 3 |
| **Met station** | Vantage Pro2 weather station, Davis Instruments | air temperature, barometric pressure, dew point temperature, rainfall, wind speed, wind direction | 2, 5 |
| **Lysimeter** | Drainage lysimeter | storage, drainage, ET | -0.62 |
| **θ_ly_ & θ_unly_** | 10HS and ECH_2_O EC-5, S-SMD-M005, Decagon Devices Inc. | soil moisture content | -0.15, -0.30  -0.05, -0.10, -0.20 |
| **θ_FEF_** | 10HS ,S-SMD-M005, Decagon Devices Inc. | soil moisture content | -0.15 |
| **P** | CS106 Campbell Scientific | barometric pressure | 1 |
| **T_soil_** | S-TMB-M006 Onset Computer Corporation, Bourne, MA | soil temperature | -0.01, -0.15, -0.30  -0.05, -0.1, -0.2 |
| **PE** | A standard weather bureau Class A evaporation pan | pan evaporation | 0.12 |
| **Porometer** | SC-1 leaf porometer, Decagon Devices Inc. | Stomatal resistance | - |
| **LAI** | AccuPAR LP-80 ceptometer, Decagon Devices Inc. | Leaf Area Index | - |

| LAS | The Scintec BLS-900 LAS | turbulent flux | 1.8 |
| --- | --- | --- | --- |
| CR1000 | CR1000 Campbell Scientific | data logger for EC data | - |
| HOBO U30 | HOBO U30 station , Onset Computer Corporation, Bourne, MA | data logger for soil moisture | - |
| HOBO Micro | HOBO Micro station, Onset Computer Corporation, Bourne, MA | data logger for soil moisture | - |
| HOBO 4 ext | HOBO 4 ext channels , Onset Computer Corporation, Bourne, MA | data logger for soil temperature | - |
